# Supplementary figures and images for: Identification of novel prophage regions in Xenorhabdus nematophila genome and gene expression analysis during phage-like particle induction
Source: PeerJ. 2022 Feb 15;10:e12956. doi: 10.7717/peerj.12956 (PMC8855722; doi:10.7717/peerj.12956)

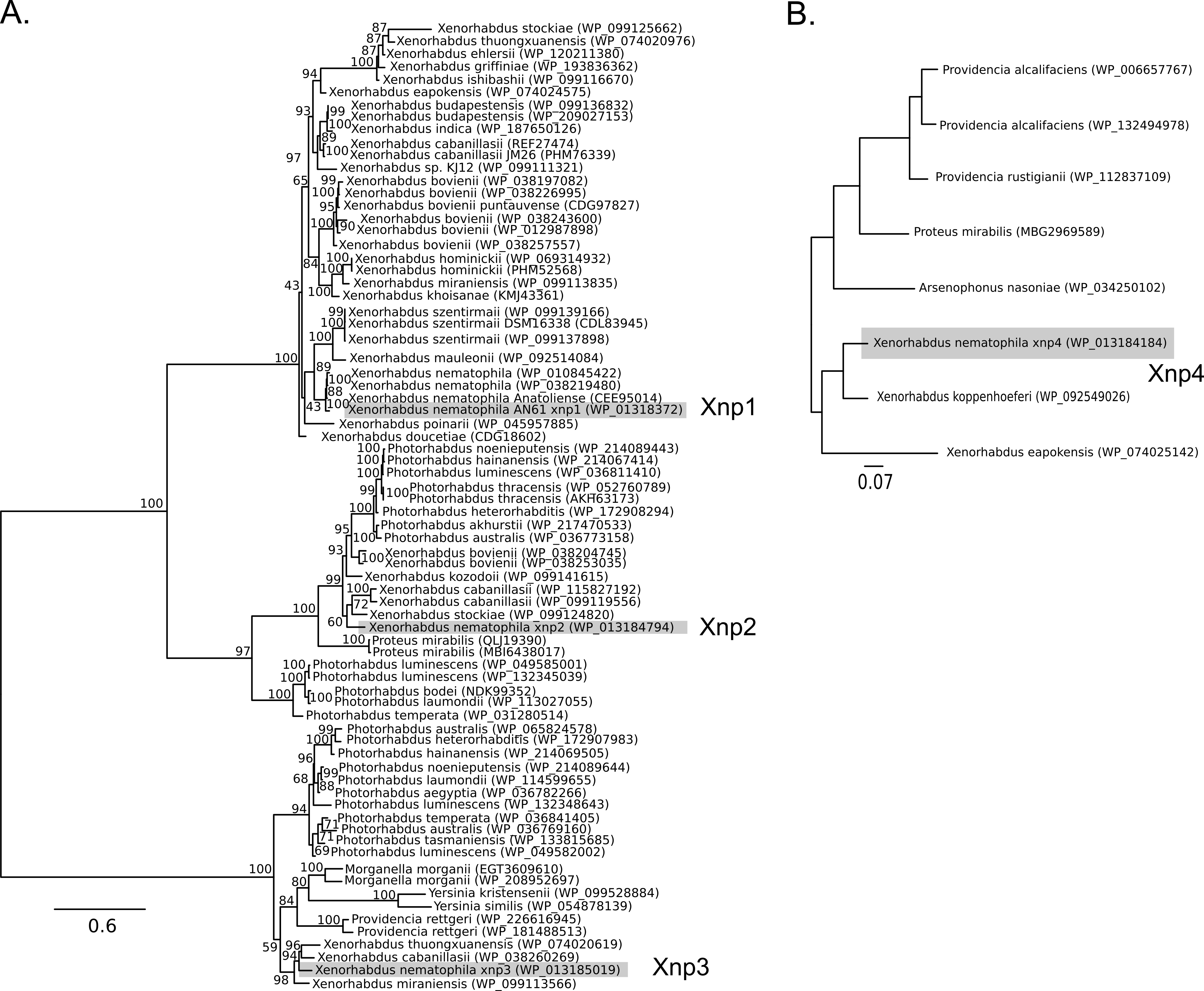

Supplement: Supplemental Information 8 — (A) Phylogeny of Phage-related minor tail protein present in Xnp1, Xnp2 and Xnp3. Analysis of 76 sequences with 1,919 amino-acid sites. (B) Phylogeny of 3 proteins (tail protein and fiber protein) present in Xnp4 prophage region and orthologous proteins present in other bacteria. Analysis of 8 sequences with 1,286 amino-acid sites. The phylogenetic analyses were inferred using ML inference using iq-tree. Nodes are associated with bootstrap values based on 1,000 replicates. The best-fit model calculated using ModelFinder according to the AIC index : JTT+F+I+G4 for A and LG+F+G4 for B. The accession number of the studied proteins are indicates in bracket after the species names. The scale bar indicates the distance in substitutions per nucleotide. [file peerj-10-12956-s008.png]
